# A Novel Bird-Shape Broadband Piezoelectric Energy Harvester for Low Frequency Vibrations

**DOI:** 10.3390/mi14020421

**Published:** 2023-02-10

**Authors:** Han Yu, Xiaofan Zhang, Xiaobiao Shan, Liangxing Hu, Xingxu Zhang, Chengwei Hou, Tao Xie

**Affiliations:** 1 State Key Laboratory of Robotics and System, Harbin Institute of Technology, Harbin 150001, China; 2 School of Electrical and Electronic Engineering, Nanyang Technological University, Singapore 639798, Singapore; 3 Ministry of Education Key Laboratory of Micro and Nano Systems for Aerospace, Northwestern Polytechnical University, Xi’an 710072, China

**Keywords:** energy harvester, low frequency, rotating motion, multimode

## Abstract

This work presents a novel bird-shaped broadband piezoelectric energy harvester based on a two-DOF crossed beam for low-frequency environmental vibrations. The harvester features a cantilever mounted on a double-hinged beam, whose rotating motions effectively diminish its natural frequencies. Numerical simulation based on the finite element method is conducted to analyze the modal shapes and the harmonic response of the proposed harvester. Prototypes are fabricated and experiments are carried out by a testing system, whose results indicate a good agreement with the simulation. The multi-frequency energy harvesting is achieved at the first-, second-, and fifth-order resonances. In particular, the proposed harvester demonstrates the remarkable output characteristics of 9.53 mW and 1.83 mW at frequencies as low as 19.23 HZ and 45.38 Hz, which are superior to the majority of existing energy harvesters. Besides, the influences of key parameters on the harvesting performance are experimentally investigated to optimize the environmental adaptability of the harvester. This work provides a new perspective for efficiently harvesting the low-frequency vibration energy, which can be utilized for supplying power to electronic devices.

## 1. Introduction

In the past few years, the technology of harvesting energy from the ambient natural environment vibration has attracted more and more interest, primarily due to the development of Micro-electro-mechanical systems [1,2]. Many microelectronic devices and sensor networks, such as implanted medical machines and embedded sensors, are inconvenient to replace their batteries, so energy supply technology is needed to provide them with a reliable and continuous energy supply to overcome the limitations. The commonly used approaches for harvesting ambient vibration energy include triboelectric, electromagnetic, electrostatic [3], and piezoelectric energy harvesting. The merits of simple structure, large power density, and simple operation mechanisms make piezoelectric energy harvesters (PEHs) widely employed in the ocean [4], pavement [5,6,7], and vibration energy harvesting [8,9,10]. Research into transducer materials has laid the foundations for the emergence of PEHs [11] The principle of the piezoelectric energy harvesting technology [12] has been studied for many years [13,14], and a certain research outcome has been achieved [15]. However, most PEHs can only produce large power output at their resonant frequencies. Their power output will decrease apparently with a slight frequency shift, which limits their applications [16].

So far, various methods have been proposed to increase the power output and extend the bandwidth of PEHs [17]. Optimizing the structure of the PEHs is a kind of effective approach to extending the working bandwidth [18]. For example, Zhang et al. [19] achieved broadband by adjusting the key structural parameters of the PEH which is composed of a matrix of piezoelectric bimorphs connected by springs. It is considered an effective way to apply the auxetic structure to the metal substrate of the PEHs [20,21]. The characteristics of the auxetic structure, such as negative Poisson’s ratio and stress concentration, can effectively increase the power output of the PEH and reduce its operating frequency [22]. However, the load capacity of the PEH will be lower accordingly. Liao et al. [23] designed a nonlinear piezoelectric energy harvester that adopted a clamp-clamp beam with auxetic structures, which could improve the efficiency and bandwidth of energy harvesting based on a purely mechanical structure. Eghbali et al. [24] proposed a new concept to enhance the efficiency of the vibration energy harvesting via the auxetic structures, which could increase the extracted power by factors of 3.9 and 7.0 for the two proposed geometries, comparing with the case in which the PZT is straightly attached to the cantilever. Introducing a multi-stable system into PEHs can also significantly improve performance [25,26,27]. Inspired by the rapid shape transition of the Venus flytrap, Zuo et al. [28] presented a bi-stable piezoelectric energy harvester, which took advantage of the mutual self-constraint at the free ends of two cantilever sub-beams with a pre-displacement, can attain broadband energy harvesting. Xu et al. [29] presented a bistable piezoelectric energy harvester dedicated to vibration energy harvesting in the direction of gravity. The frequency range with over 30% peak value of the average output power was around 16 to 20.4 Hz. Most multi-stable piezoelectric energy harvesters perform poorly at relatively low excitation levels, and their inter-well chaotic vibration is hard to avoid, which increases the difficulty of collecting electricity [30].

Because the environmental natural frequencies are lower in general, researchers proposed many structures to harvest energy at low frequencies. Atmeh et al. [31] designed a frequency up-conversion PEH which uses a magnetic coupling structure to convert lower frequency vibrations into higher frequency vibrations. Fu et al. [32] also proposed a nonlinear pendulum created by the magnets to achieve working at ultra-low frequency environment. Though these existing PEHs could effectively harvest energy at low frequencies, their output power is undesirable in general. Because of the electromagnetic interference generated by the magnets, the appeal structure has a very limited environment for use.

In recent years, researchers have applied multi-degree-of-freedom (DOF) systems to piezoelectric energy harvesters to widen the operating bandwidth by adjusting two or more resonance frequencies. The piezoelectric cantilever array is a typical multi-DOF system, and different resonant frequencies can be obtained by adjusting the size of the cantilever beam and the tip masses. Dai et al. [33] proposed a multi-DOF wideband PEH consisting of five tip masses, two U-shaped cantilever beams, and a straight beam with five resonant frequencies [34], which can effectively enlarge the operating bandwidth and collect vibration energy in low frequencies [35,36,37]. Ding et al. [38] proposed a variant power generator array using an arc-shaped piezoelectric cantilever beam array, which had a satisfactory output performance. However, these designs can be regarded as combinations of multiple harvesters with different natural frequencies. If one of the cantilevers vibrates at its natural frequency, the other has almost no power output, which decreases the power density vastly. To address the above issues, researchers applied the coupling effect to the piezoelectric beam array. Lan et al. investigated a dual-beam vibration energy harvester in which two piezoelectric cantilever beams are coupled by magnets. The magnetic interaction can achieve high-energy oscillations for both beams to achieve power enhancement. Shim et al. designed a nonlinear piezoelectric energy harvester with a coupled beam array consisting of a base, two elastic supports, and four piezoelectric beams with different natural frequencies. The four beams had relatively high output power at frequencies from 40 to 80 Hz. However, these designs often only use the first natural frequency of the beam because their second natural frequency is too high to be effectively used. Besides, most vibration energy sources in the natural environment have low frequencies, so it is essential to reduce the operating frequency of multi-degree-of-freedom piezoelectric energy harvesters [39].

In summary, the existing multi-DOF wideband PEHs have some drawbacks such as low power density, the presence of electromagnetic interference, and complex structures. Therefore, this paper proposes a bird-shaped two-DOF piezoelectric energy harvester based on a crossed beam with rotating motions to enhance the low-frequency vibration energy harvesting performance and to expand the operating bandwidth. The structure reduces its higher-order natural frequencies by means of a double-hinged support beam, eliminating the need for magnets and effectively avoiding electromagnetic interference. The simple and compact structure also facilitates use in arrays or uses in largely concentrated arrangements. The contributions of this work are as follows: (1) Inspired by the flapping wings of birds, a novel PEH based on a two-DOF crossed-beam structure is proposed to achieve broadband energy harvesting. (2) The crossed beam features a cantilever mounted on a double-hinged beam, whose rotating motions effectively reduce the second-order natural frequency of the system and enhance the harvesting performance at low frequencies. (3) This work can achieve an awe-inspiring output power, which can effectively power the microelectronic devices and sensor networks. (4) This work provides a new idea for the design of PEHs.

## 2. Designing Process and Working Principle of the Harvester

### 2.1. Structural Design

**Figure 1:**
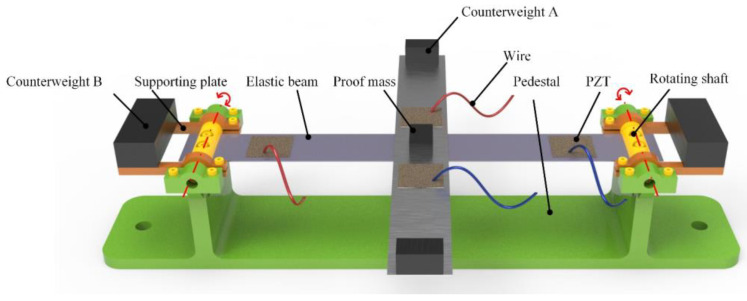
The structure and component parts of the piezoelectric energy harvester.

**Figure 2:**
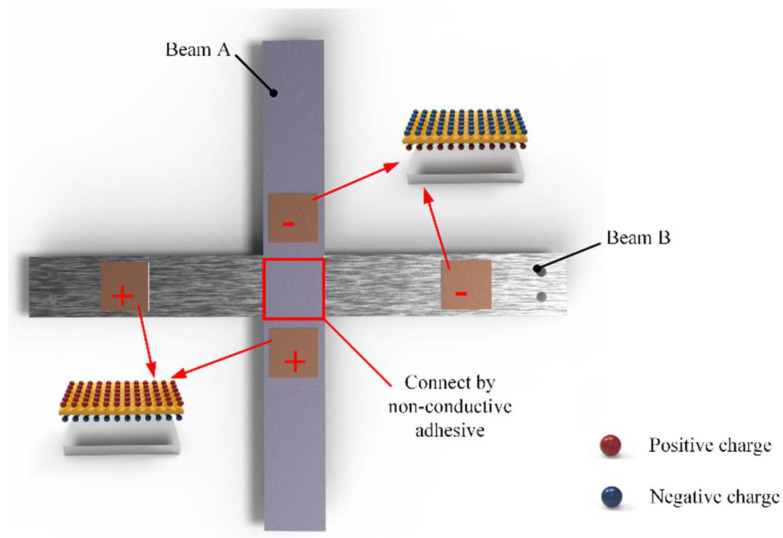
The connection style of the energy harvester.

Figure 1 shows the structural schematic of the proposed energy harvester. The energy harvester is composed of two elastic beams, the proof mass, the counterweight A and the counterweight B, PZT sheets, the pedestal, the rotating shaft, the supporting plate, and the wire. Two beams called beam M and beam W form the crossed-beam structure, as shown in Figure 2. Beam M and beam W are connected by one kind of non-conductive adhesive and every two PZT sheets are pasted symmetrically at the ends of each beam in series. In other words, beam M and beam W are two independent power supplies.

The ends of beam W are hinged by two rotating shafts and the supporting plates are fixed on the other sides of the rotating shafts as shown in Figure 1. Counterweight B which is used to enhance the output characteristics of the harvester is mounted on the supporting plate. To further enlarge the vibrational response, the proof mass and counterweight A must adhere to the center and the end of beam M, respectively. The employment of the rotating shaft effectively reduces the natural frequency of the harvester and enhances its environmental adaptability. It is worth noting that counterweights with different specific gravities can adjust the output voltage of the energy harvester at different frequencies. More details will be illustrated in Section 5.

### 2.2. Working Principle

Unless otherwise specified, the output of beam M and beam W described below represent the output of PZT sheets in series on each beam, respectively. Compared with the cantilever-type piezoelectric energy harvester, the adoption of the hinged supporting boundaries greatly reduces its first- and fifth-order natural frequencies. It is worth noting that this low-frequency harvesting technology does not degrade its outputs. At present, the output power of a low-frequency energy harvester is not ideal, which is mostly at the μW level. However, the output power of this bird-shaped energy harvester can reach the mW level. The energy harvester in this work can effectively harvest energy at its first, fourth, and fifth natural frequencies. The counterweight A and the proof mass are used to amplify the strain in PZT sheets. When the environmental frequency is close to its resonant frequency, the harvester vibrates according to its modal shapes. The deformation of the beam leads to a gradual increase in the strain in PZT sheets. According to the direct piezoelectric effect, positive and negative charges will be generated on the surfaces of PZT sheets.

## 3. Finite Element Method

### 3.1. Finite Element Modeling

**Figure 3:**
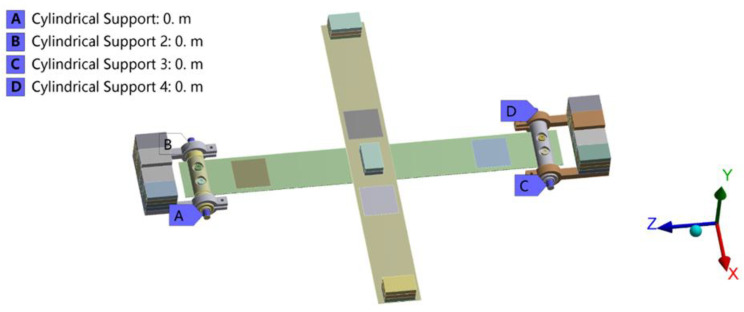
The simulation model of the energy harvester and its constraints.

The output characteristic of the energy harvester is analyzed by finite element simulation. In fact, the working process of the harvester is involved in multi-physics couplings such as mechanical and electric fields. Firstly, the modal analysis is performed to reveal its dynamic properties at different resonant frequencies. Figure 3 illustrates the simulation model of the energy harvester and its constraints. It is important to note that the bears and pedestal are ignored in the simulation for saving computing resources.

According to Figure 3, surface a, b, c, and d are constrained by the cylindrical support whose tangential direction is set to be free and other directions are fixed. To further increase the computing efficiency and improve the calculation precision, dense grids are generated in beams and PZT sheets, while sparse grids are arranged in other parts. More detailed information about the material parameters which is used in the simulation is shown in Table 1.

### 3.2. Modal Analysis

**Figure 4:**
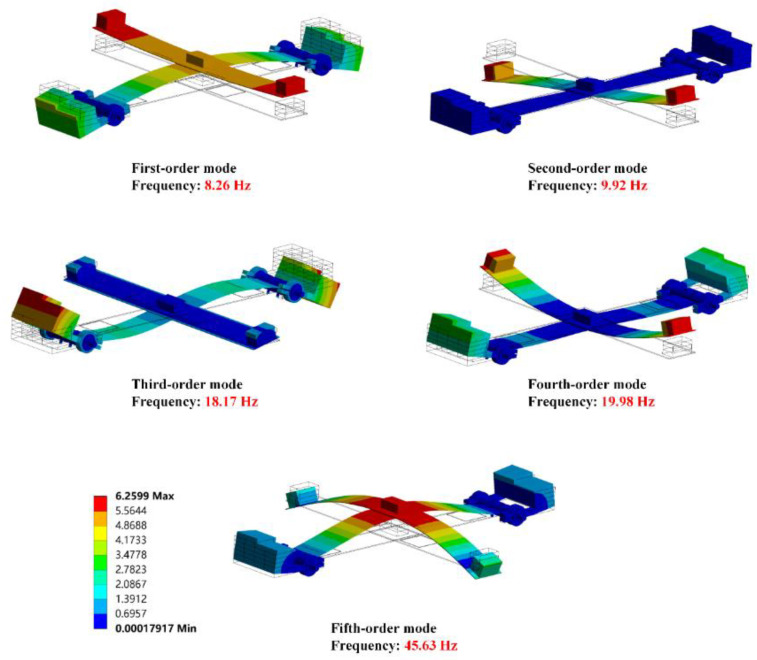
Modal analysis of the energy harvester.

Figure 4 shows the first five natural modes of the energy harvester. In this paper, the main working modes are the first-order, fourth-order, and fifth-order modes, while the output voltage in the second-order and third-order modes is very small. The reason is that the strain in PZT sheets at its second-order resonant frequency is so small that there are few induced charges. Though the PZT sheets on beam W generate a larger strain at its third-order resonant frequency, the strain phase of two PZT sheets connected in a series on beam W is reversed. Therefore, the induced charges on the surfaces of two PZT sheets cannot lead to the electric potential difference between the two wires. In summary, the energy harvester has almost no output at its second-order and third-order natural frequencies.

The deformation shapes of the effective operating modes (the first-order, the fourth-order, and the fifth-order) of the energy harvester like a bird in flight. For getting a clearer understanding of the structure, the modal shape of the energy harvester can be divided into two parts: the bird’s body (beam W) and wing (beam M). According to Figure 4, the shape of beam W is like a ‘rainbow’ at its first-order resonant frequency, and the middle of beam W is the position of maximum displacement. At the same time, counterweight A generates small vibrations up and down relative to the middle of the beam which looks akin to the wings of a bird. The vibration amplitudes of counterweight A become larger at its fourth-order natural frequency. And the position of PZT sheets is where the maximum displacement generates on beam W at its fourth-order resonant frequency. In fact, the deformation of beam M in the fifth-order mode is similar to that in the fourth-order mode, while the deformation of beam W has different patterns. The moving directions of the proof mass and counterweight B are the same at its fifth-order resonant frequency. For this reason, the fifth-order modal shape of beam W presents as a ‘W’ shape.

### 3.3. Harmonic Response Analysis

**Figure 5:**
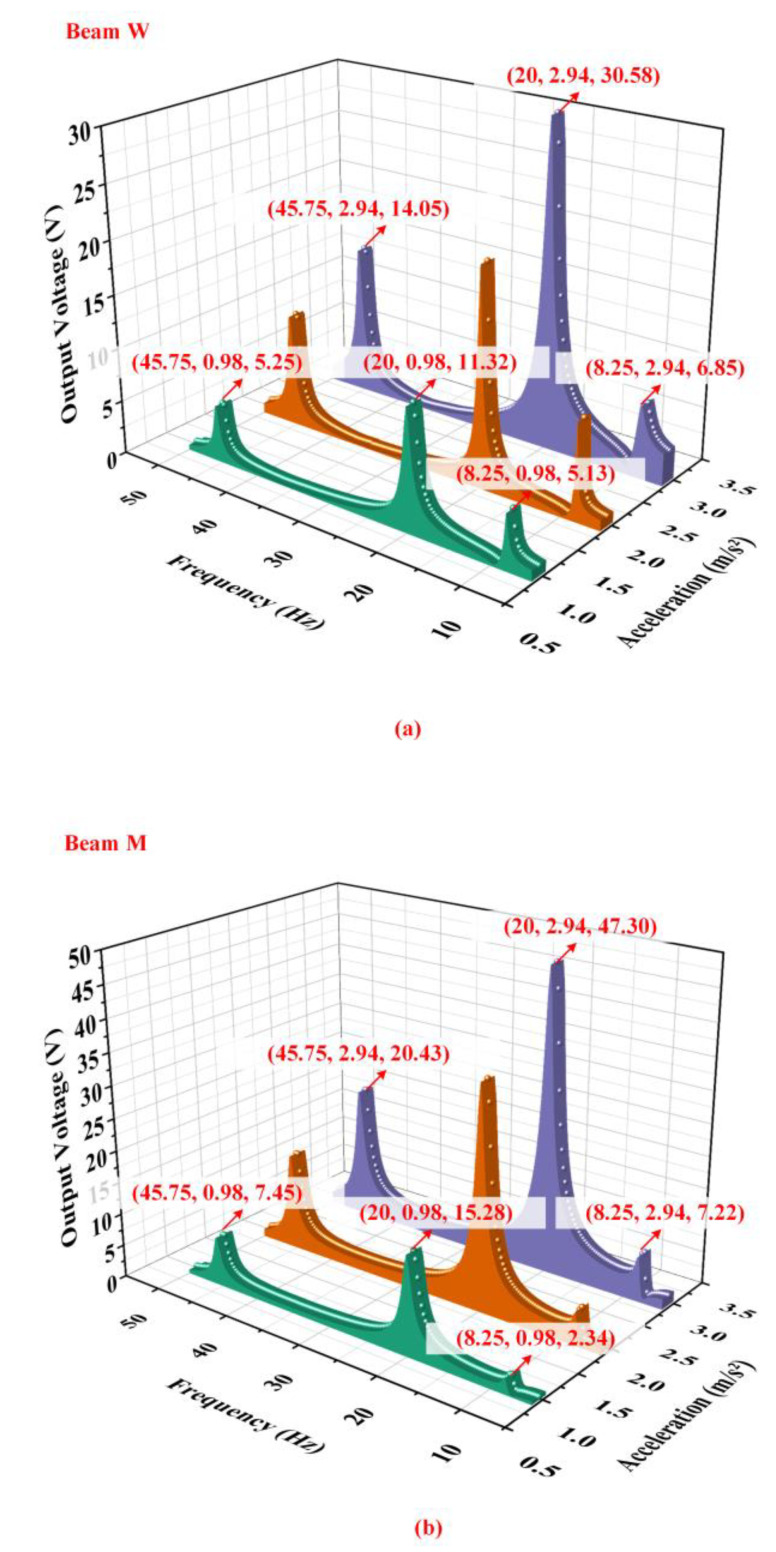
The output voltage of (**a**) beam M and (**b**) beam W with different accelerations obtained by simulation.

Figure 5 shows the output voltage of beam M and beam W with different accelerations obtained by simulations when the proof mass is 10 g, counterweight A is 15 g, and counterweight B is 90 g. According to Figure 5, it can be seen that the output characteristics of the energy harvester is excellent. When the acceleration is 2.94 ${m/s}^{2}$, the output voltage of beam W can reach 30.58 V and the output voltage of beam M can reach 47.3 V. The energy harvester has demonstrated outstanding broadband ability and brilliant output characteristics at low frequencies.

## 4. Experimental Setup

**Figure 6:**
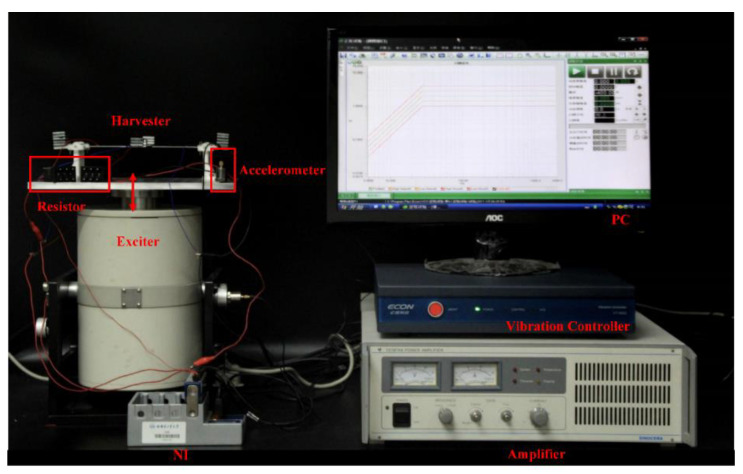
Photograph of the experimental testing system for energy harvesting.

According to Figure 6, the whole experimental system is composed of a PC, an electromagnetic exciter (JZK-50, Sinocera Piezotronics Inc., Yangzhou, China), a vibration controller (VT-9002, ECON Inc., Hangzhou, China), a power amplifier (YE5874A, Sinocera Piezotronics Inc., Yangzhou, China), a harvester prototype, a resistor, a piezoelectric accelerometer (YD4-308, Honeywell International Inc., Charlotte, NC, USA) and a data acquisition card (NI-9229, National Instruments Inc., Austin, CO, USA). The vibration controller is controlled by the software on the PC to adjust the frequencies and amplitudes of the sinusoidal signals. The signal output by the vibration controller supplies power to the electromagnetic exciter through the power amplifier to drive the prototype to vibrate. The piezoelectric layers of the prototype are connected to the resistor through wires, and the voltage across the resistor is monitored and recorded using the data acquisition card. During the process, the piezoelectric accelerometer will always record the acceleration output of the electromagnetic exciter, thus giving negative feedback to the vibration controller.

## 5. Results and Discussion

### 5.1. Effects of the Base Acceleration

**Figure 7:**
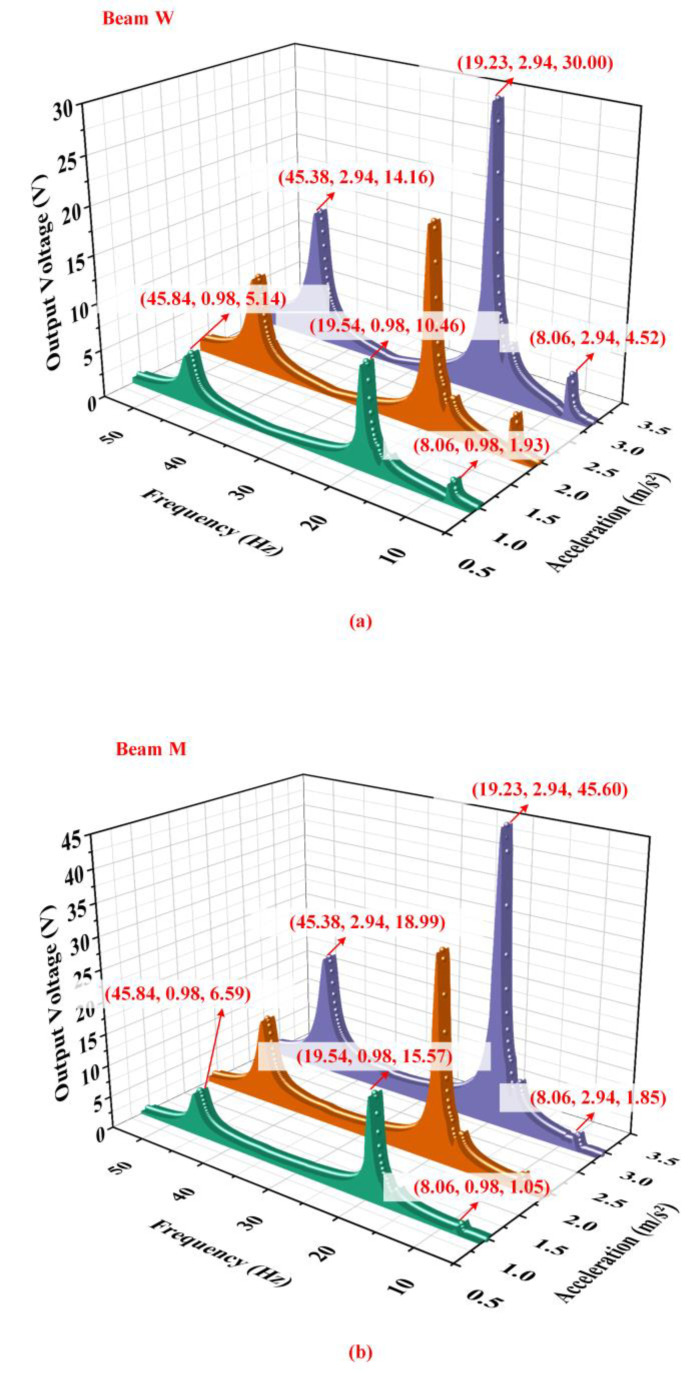
The output voltage of (**a**) beam W and (**b**) beam M under different accelerations by experiment.

**Figure 8:**
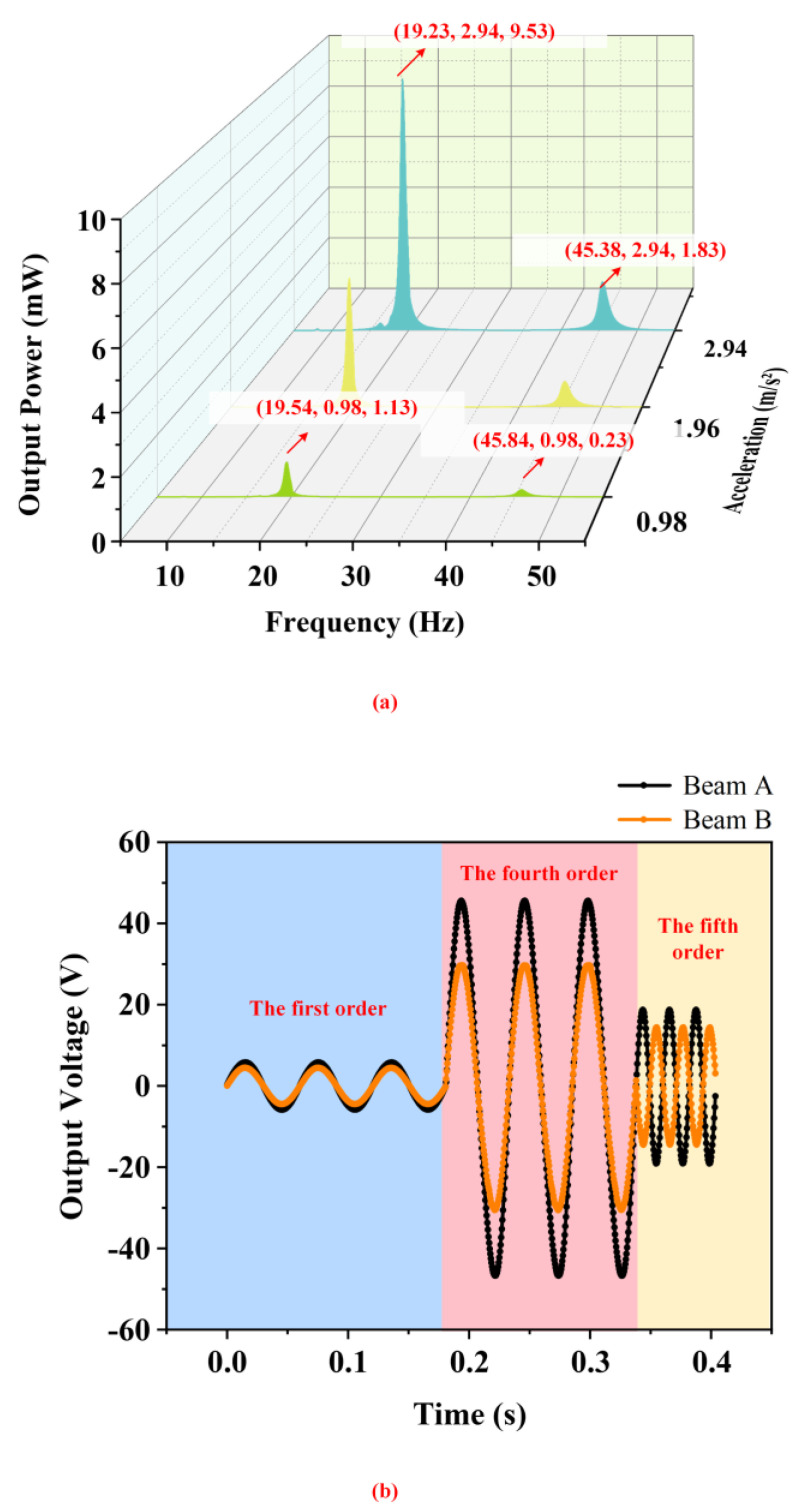
The output characteristics of the PEH under different accelerations by experiment. (**a**) The output power of the energy harvester under different accelerations; (**b**) the time−domain waveforms of beam M and beam W.

Figure 7 shows the output characteristic of the energy harvester under different accelerations when the proof mass is 10 g, counterweight A is 15 g, and counterweight B is 90 g. Figure 7a,b illustrate the influence of the acceleration on the output characteristics of the energy harvester acquired by the experimental results. It can be seen that its output voltage with an external resistance of 300 kΩ rapidly enlarges with increasing acceleration. Because of the deviation of the experiment and data processing, the resonant frequencies of the energy harvester have a few changes under different accelerations. However, the changes are so small that can be ignored. It is worth noting that beam M and beam W show excellent output properties in the fourth-order mode. On the contrary, the output of the harvester in the first-order mode is poor so it cannot play a main role in practical application. The harvester output in the fifth-order mode can meet the power supply needs of low-power equipment. The total output power of the energy harvester with an external resistance of 300 kΩ which is the sum of two sets of power supplies is shown in Figure 8a. When the base acceleration is 2.94 ${m/s}^{2}$, the total output power is as high as 9.53 mW at 19.23 Hz and 1.83 mW at 45.38 Hz, which is a considerable result in the field of piezoelectric energy harvesting. Figure 8b shows the time-domain waveforms of beam M and beam W. According to Figure 8b, the phases of output voltages of beam M and beam W are the same at the first-order and fourth-order resonant frequencies but are opposite at the fifth-order resonant frequency. In the fifth-order resonance, the upper surface of beam M is stretched while the upper surface of beam W is compressed at the same time. For this reason, the phases of the output voltage of two beams in the fifth-order resonance are opposite.

### 5.2. Effects of the Proof Mass

**Figure 9:**
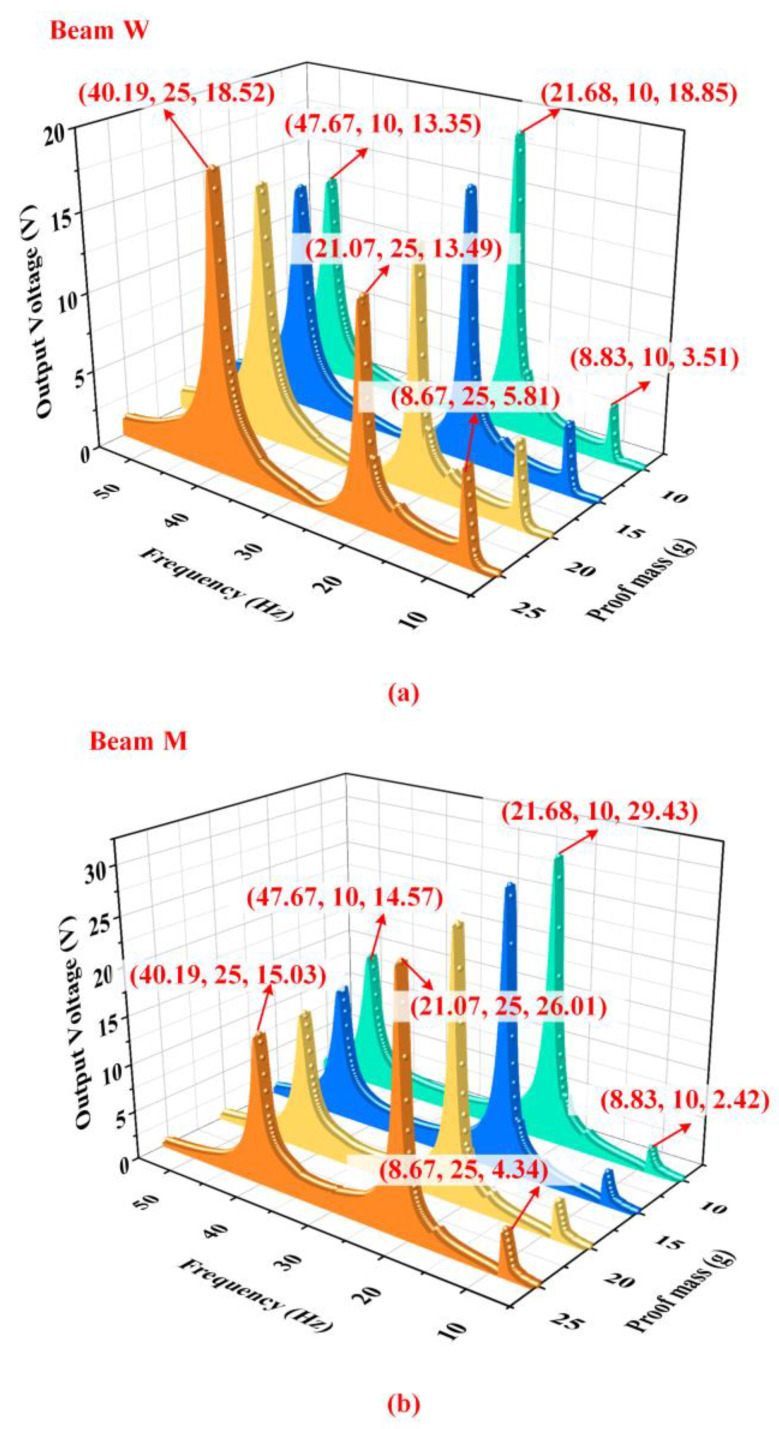
The output voltage of (**a**) beam W and (**b**) beam M with different weights of the proof mass by experiment.

Figure 9 shows the influence of different weights of the proof mass on the output characteristics of the energy harvester when counterweight A is 15 g, counterweight B is 60 g and acceleration is 1.96 ${m/s}^{2}$. According to Figure 9a, the output characteristics of beam W have regular changes with different weights of the proof mass. When the weight of the proof mass was added, the output voltage of beam W in the first-order and fifth-order modes significantly increases while the output voltage in the fourth-order mode reduces. This phenomenon is attributed to the deformation characteristics of the harvester under different natural modes. Figure 4 illustrates that the proof mass generates large displacements at its first-order and fifth-order resonant frequencies. Since both ends of beam W are constrained by hinges, the strain in the PZT sheets of beam W will enlarge with the increasing displacement of the proof mass, which can be accomplished by increasing the weight of the proof mass. According to the direct piezoelectric effect, the output voltage will also have significant enlargement.

**Figure 10:**
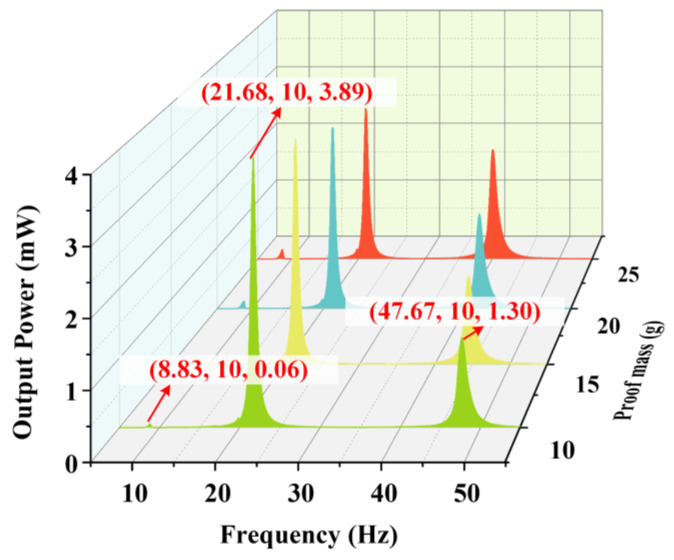
The output power of the energy harvester with different weights of the proof mass.

There is a difference in the moment of inertia between the proof mass and counterweight B leading to a hysteresis effect, which makes their moving directions opposite. The difference in the moment of inertia decreases with the increasing of the weight of the proof mass, so the rotating angle of the shaft became small at the fourth-order resonant frequency due to the attenuation of the hysteresis effect. Therefore, when the weight of the proof mass increases, the strain of the PZT sheets on beam W decreases, which leads to the decline in the output voltage of beam W in the fourth-order resonance. It can be seen from Figure 9b that the change of the proof mass does not have a noticeable effect on the output voltage of beam M. The reason is that there are few influences of the proof mass on the fourth-order mode of the energy harvester. Figure 10 shows the total output power of the harvester at 1.96 ${m/s}^{2}$. To sum up, the energy harvester should select the specific weight of the proof mass according to its working environment.

### 5.3. Effects of Counterweight A and Counterweight B

**Figure 11:**
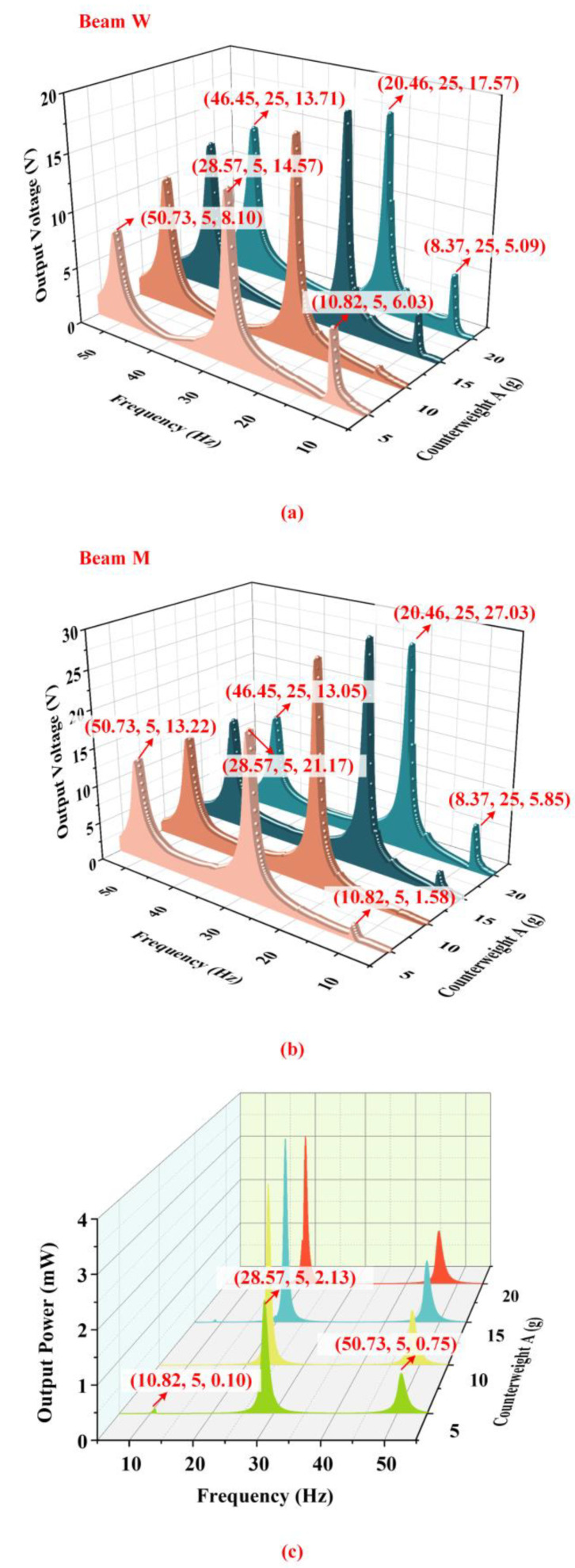
The output characteristics of the energy harvester with different weights of counterweight A. (**a**) The output voltage of beam W; (**b**) The output voltage of beam M; (**c**) The total output power of the harvester at 1.96 ${m/s}^{2}$.

Figure 11 shows the output characteristics of the energy harvester with different weights of counterweight A when the proof mass is 10 g, counterweight B is 60 g and the acceleration is 1.96 ${m/s}^{2}$. According to Figure 11a,b, the changes of counterweight A have bigger influences on the output of beam M than that of beam W. The output voltage of beam M in the first-order and fourth-order modes enhances with the increasing of the weight of counterweight A while the output of beam M in the fifth-order mode has almost no change in this process. Figure 4 could explain the fundamental principle of harvesting performance. Counterweight A at the ends of beam M occurs large displacements at the first-order and fourth-order resonant frequencies, which is the key factor leading to the deformation of the PZT sheets. Beam M presents a ‘bow’ shape at the first-order and fourth-order resonant frequencies. When the weight of counterweight A increases, the strain of the PZT sheets is enhanced so the output voltage of beam M in the first-order and fourth-order modes increases. Nevertheless, counterweight A is not the key factor influencing the output of beam M in the fifth-order mode. The output of beam W in the fifth-order mode is enhanced with the increase in counterweight A because the inertial force at the position of the proof mass is enhanced. However, the change in counterweight A has few effects on the output of beam W in the first-order and fourth-order modes.

**Figure 12:**
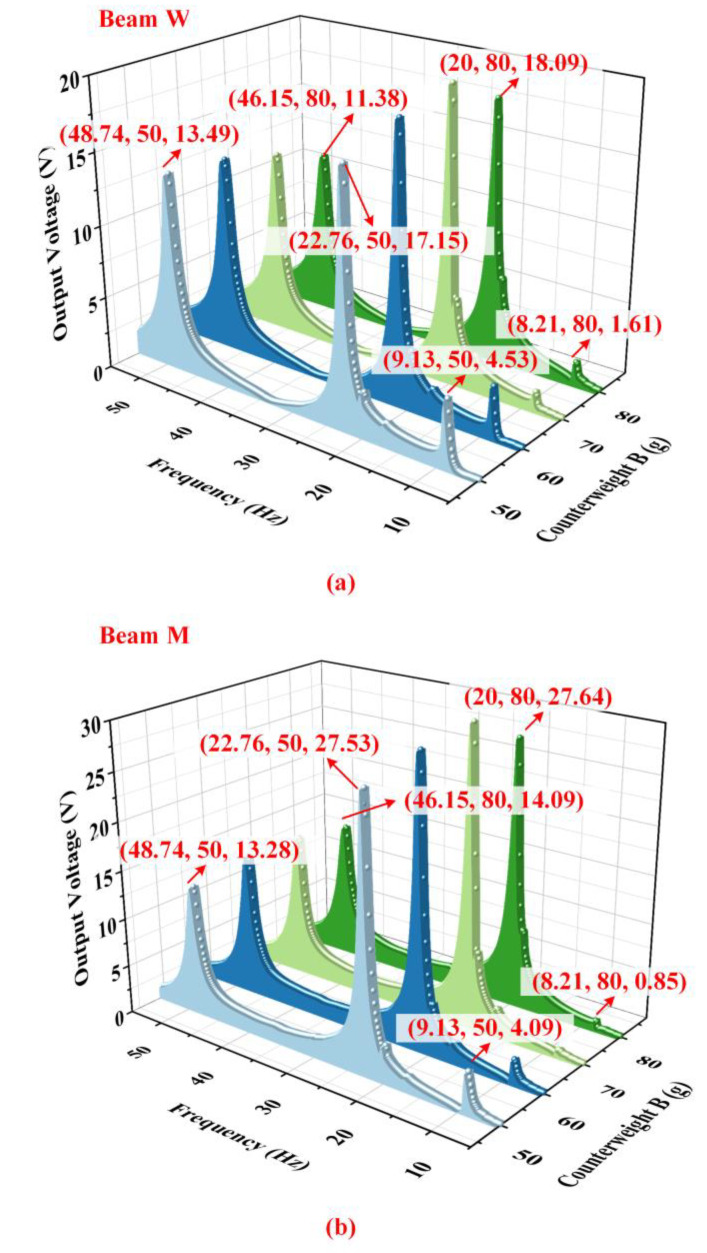
The output voltage of (**a**) beam W and (**b**) beam M with different weights of counterweight B.

**Figure 13:**
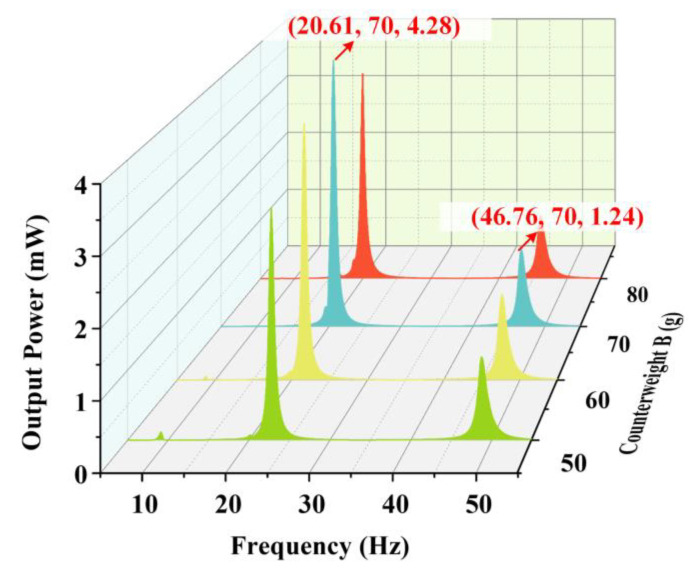
The total output power of the harvester with different weights of counterweight B.

Figure 12 shows the influence of different weights of counterweight B on the output characteristics of the harvester when the proof mass is 10 g, counterweight A is 15 g, and the acceleration is 1.96 ${m/s}^{2}$. According to Figure 12a,b, it can be seen that the influence of the counterweight on the output voltage is negligible. However, the natural frequencies of the harvester gradually decrease due to the increase in counterweight B. It is worth noting that the harvester output can be as high as 4.28 mW at 20.61 Hz and 1.24 mW at 46.76 Hz when the counterweight B is 70 g, as shown in Figure 13.

### 5.4. Discussion and Application

According to Table 2, it can be seen that current low-frequency energy harvesters generally have the problem of low output power density such as the harvester proposed by Wang et al. [40] and Wang et al. [41]. While the PEH [42] could generate ideal power density, its structure is quite complex, and it is difficult to use for forming arrays. And only one beam of the multi-frequency energy harvester [42] vibrates at its resonant frequency because it lacks the coupling between beams. Therefore, a kind of low-frequency multi-frequency PEHs which could output high power density and have compact and stable construction is desperately needed. In this work, two independent power supplies with compact structures can be operated simultaneously at very low resonant frequencies and have high output power.

## 6. Conclusions

In this work, a novel bird-shaped piezoelectric energy harvester was presented for multi-frequency and low-frequency environmental vibration energy harvesting. The harvester was composed of a cantilever and a double-hinged beam, which formed a crossed-beam structure. Finite element analysis was carried out to estimate the deformation characteristics and output voltages of the harvester at its resonant frequencies. Harvester prototypes were manufactured to conduct experimental validation in our established testing system. The results of the experiments were in accord with those of the simulation.

Its first five orders of natural frequency were down to the effective operating range, due to the use of double-hinged support and the arrangement of the crossbeam. The first-order, second-order, and fifth-order resonant frequencies were 8.06 Hz, 19.23 Hz, and 45.38 Hz, respectively (at which the harvester had an obvious output response). Especially, the output power was as high as 9.53 mW at 19.23 Hz and 1.83 mW at 45.38 Hz which is superior to most of the previously reported harvesters. And the designed PEH avoided generating electromagnetic interference by no magnets used. This simple and compact construction not only increased the output power but was also more reliable and more suitable for array use. Moreover, the effects of the excitation acceleration, the proof mass, and the counterweight on the harvesting performance were further investigated by experiments. By changing these structural parameters, the output power and natural frequencies would be adjusted, which enhanced the environmental adaptability of the harvester. The presented harvester demonstrates excellent performance for harvesting environmental vibration energy of low frequencies, which provides a fine application prospect for supplying power to electronic devices.

## Figures and Tables

**Table 1:** Material and structural parameters of the energy harvester.

Parameters	Units	Values
The density of beam M and beam W	$\mathrm{kg}/m^{3}$	2770
Young’s Modulus of beam M and beam W	GPa	71
Poisson’s Ratio of beam M and beam W	------	0.33
The thickness of the beam M and beam W	mm	0.6
Width of the beam M and beam W	mm	25
Density of shaft	$\mathrm{kg}/m^{3}$	1130
Young’s Modulus of shaft	MPa	2589
Poisson’s Ratio of shaft	------	0.4
Density of counterweight	$\mathrm{kg}/m^{3}$	8000
Young’s Modulus of counterweight	GPa	200
Poisson’s Ratio of counterweight	------	0.3
The density of PZT sheets	$\mathrm{kg}/m^{3}$	7500
Piezoelectric tensor	$C/m^{2}$	$\left[\begin{array}{cccccc} 0 & 0 & 0 & 0 & 10.5 & 0\\ 0 & 0 & 0 & 10.5 & 0 & 0\\ -4.1 & -4.1 & 14.1 & 0 & 0 & 0 \end{array}\right]$
Stiffness tensor	GPa	$\left[\begin{array}{cccccc} 132 & 73 & 71 & 0 & 0 & 0\\ 73 & 115 & 73 & 0 & 0 & 0\\ 71 & 73 & 132 & 0 & 0 & 0\\ 0 & 0 & 0 & 26 & 0 & 0\\ 0 & 0 & 0 & 0 & 26 & 0\\ 0 & 0 & 0 & 0 & 0 & 30 \end{array}\right]$
Permittivity tensor	------	$\left[\begin{array}{ccc} 804 & 0 & 0\\ 0 & 804 & 0\\ 0 & 0 & 660 \end{array}\right]$
The thickness of PZT sheets	mm	0.3
Width of PZT sheets	mm	20
Length of PZT sheets	mm	20
Length of beam M	mm	200
Length of beam W	mm	220

**Table 2:** Comparison of the harvesting performance of this work with the other researches.

Reference	Piezoelectric Material	Dimension of Piezoelectric Materials (mm)	Resonant Frequency (Hz)	$\mathrm{Output}\;\mathrm{Power}\;\mathrm{Density}\;(\mu W/mm^{3})$	$\mathrm{Acceleration}\;(m/s^{2})$	Configuration
Wang et al. [40]	PZT	30 × 6 × 1	52	0.42	---	** 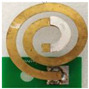 **
Wang et al. [41]	PZT	22 × 19.5 × 0.35	10	1.45	4.9	** 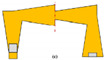 **
Huang et al. [42]	PZT	30 × 20 × 0.2	1–15	11.08	0.98	** 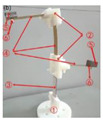 **
This work	PZT	20 × 20 × 0.3	19.23	19.85	2.94	** 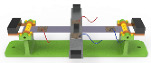 **
			45.38	3.81		

## Data Availability

The data of this work are available from the corresponding author upon reasonable request.
